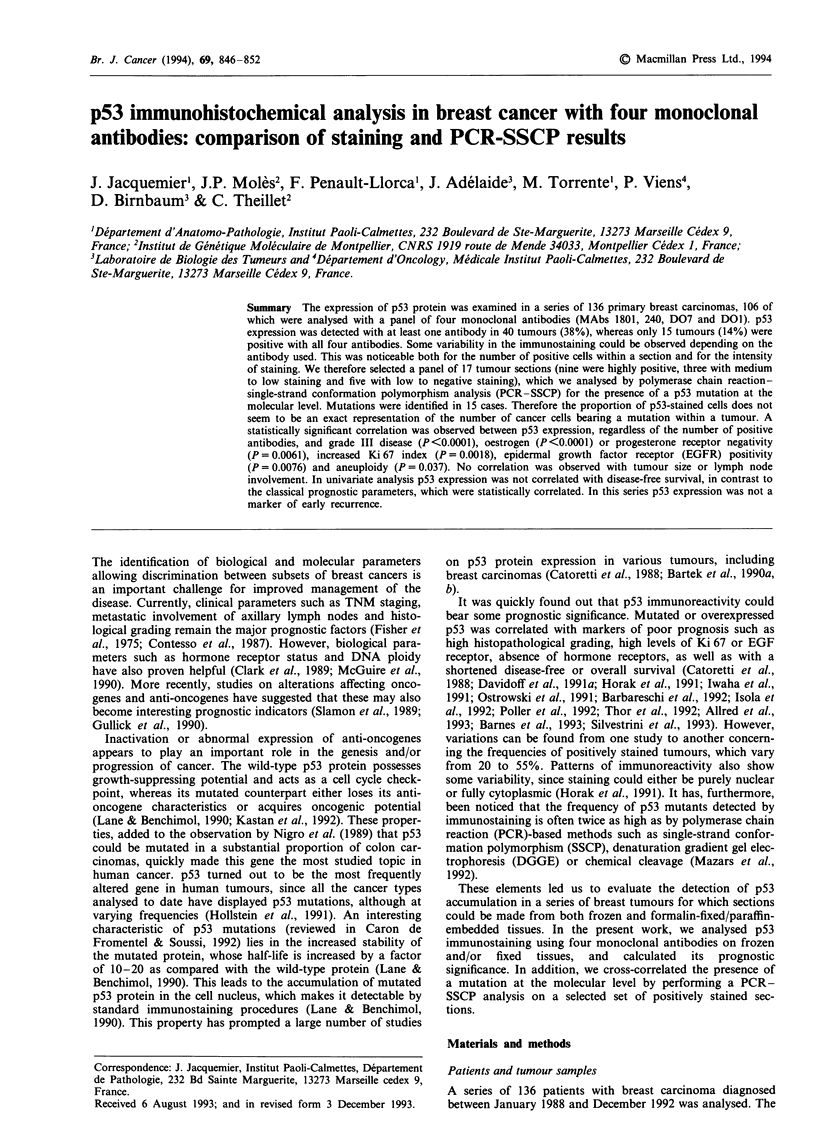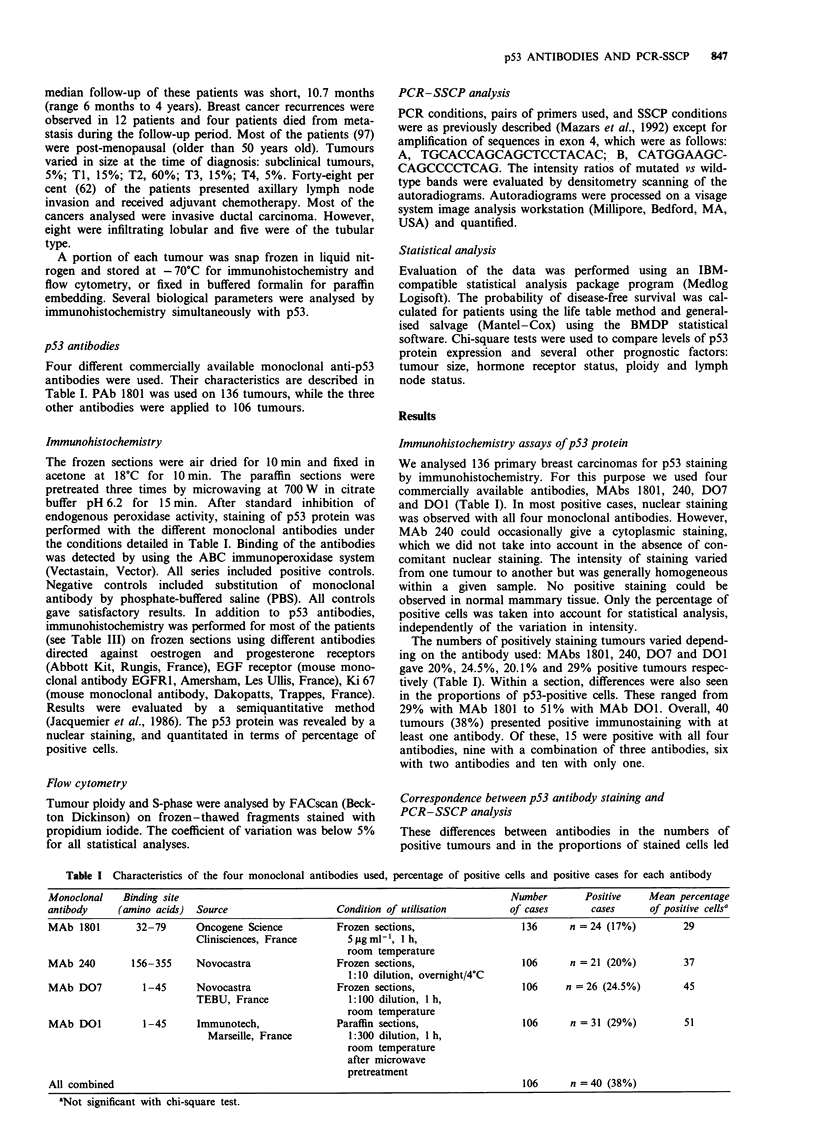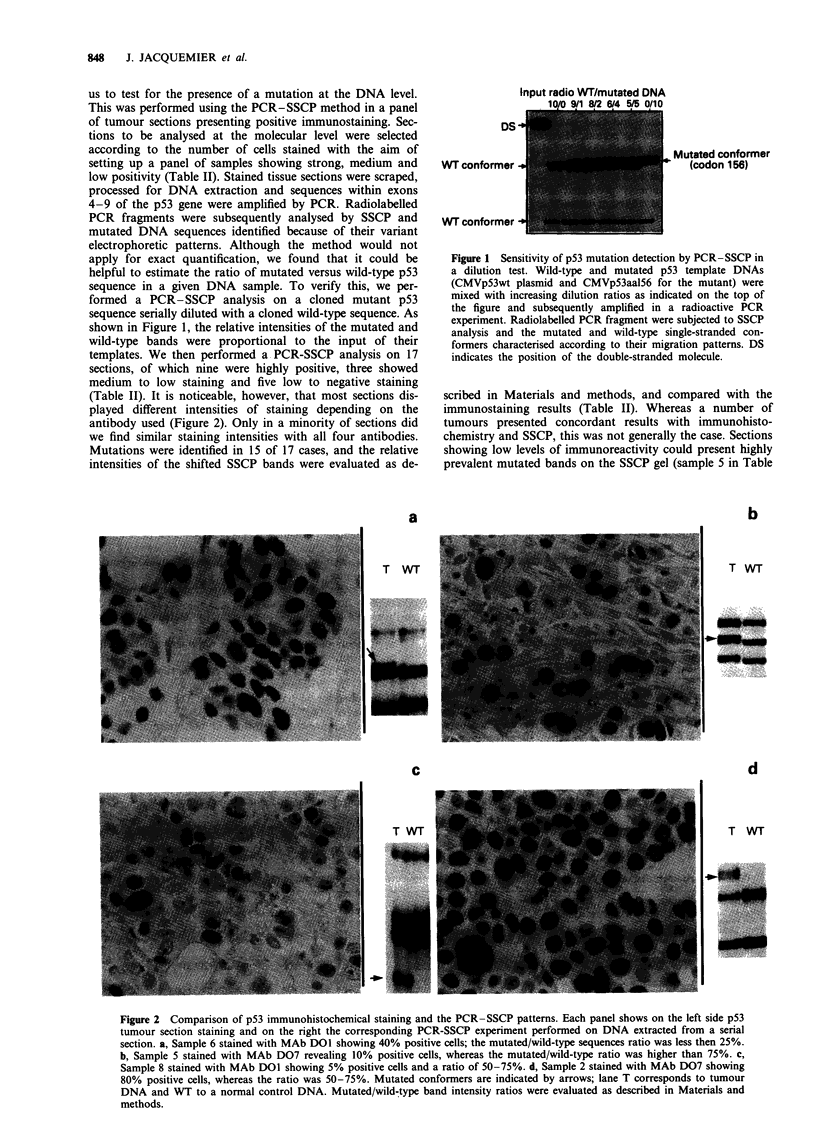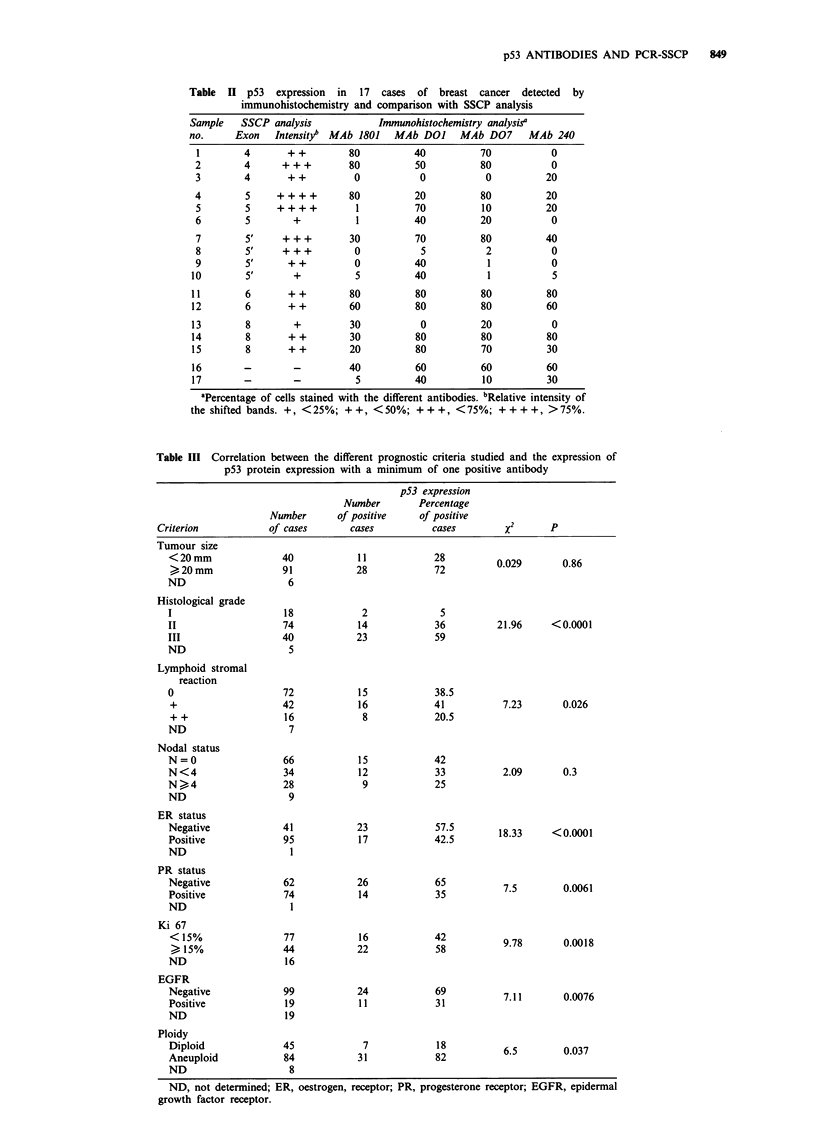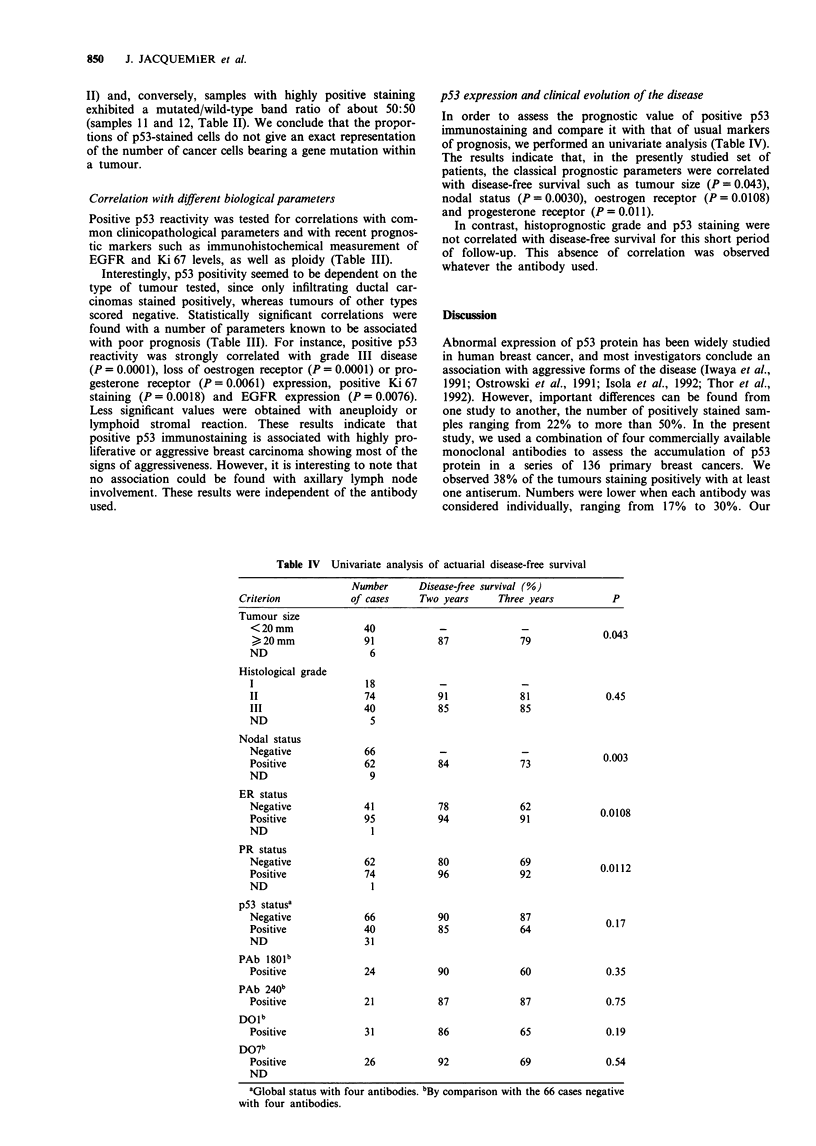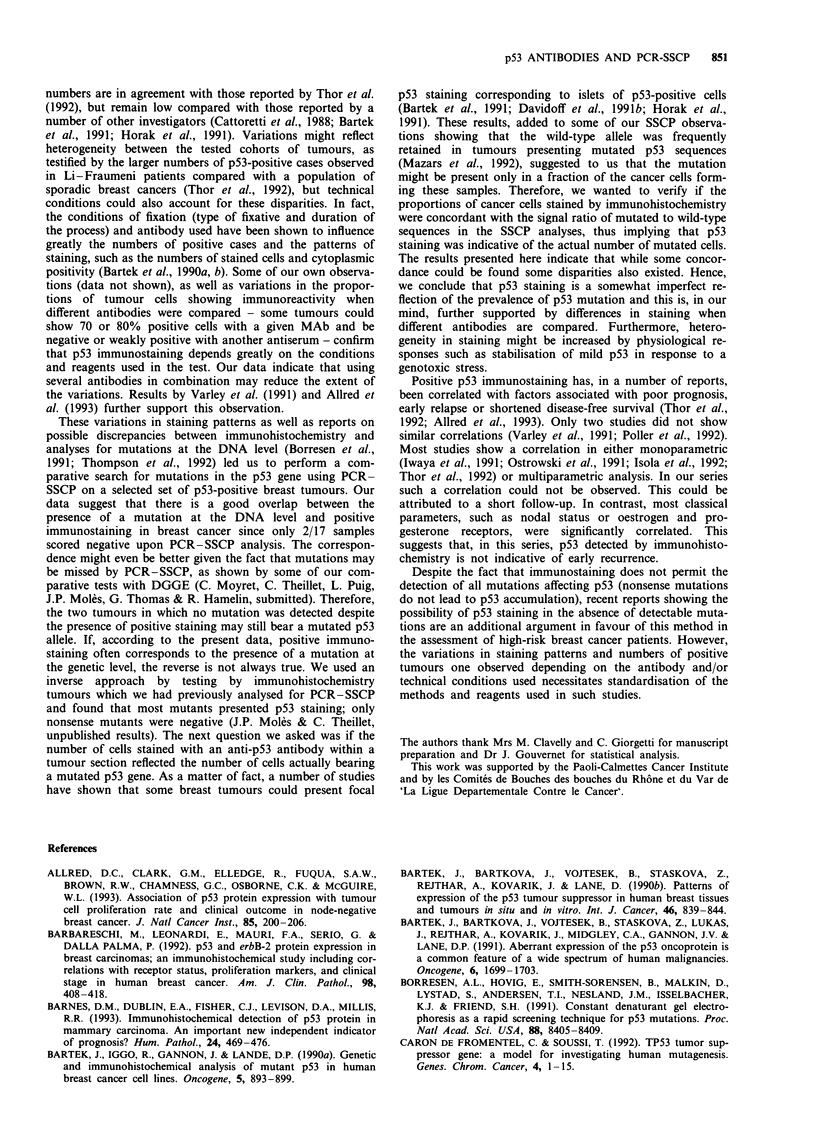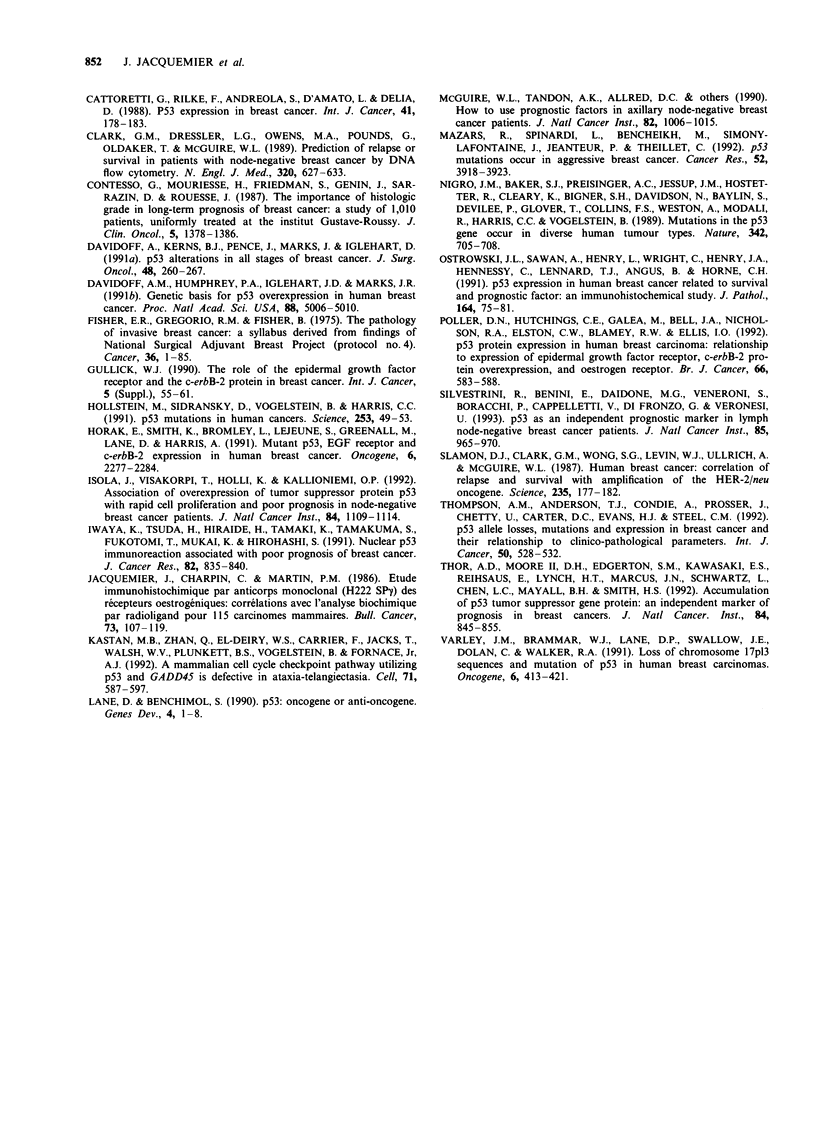# p53 immunohistochemical analysis in breast cancer with four monoclonal antibodies: comparison of staining and PCR-SSCP results.

**DOI:** 10.1038/bjc.1994.164

**Published:** 1994-05

**Authors:** J. Jacquemier, J. P. Molès, F. Penault-Llorca, J. Adélaide, M. Torrente, P. Viens, D. Birnbaum, C. Theillet

**Affiliations:** Département d'Anatomo-Pathologie, Institut Paoli-Calmettes, Marseille, France.

## Abstract

**Images:**


*Br. J. Cancer* (1994), **69**, 846–852 © Macmillan Press Ltd., 1994

### p53 immunohistochemical analysis in breast cancer with four monoclonal antibodies: comparison of staining and PCR-SSCP results

J. Jacquemier[1], J.P. Molès[2], F. Penault-Llorca[1], J. Adélaide[3], M. Torrente[1], P. Viens[4], D. Birnbaum[3] & C. Theillet[2]

[1]*Département d'Anatomo-Pathologie, Institut Paoli-Calmettes, 232 Boulevard de Ste-Marguerite, 13273 Marseille Cédex 9, France;* [2]*Institut de Génétique Moléculaire de Montpellier, CNRS 1919 route de Mende 34033, Montpellier Cédex 1, France;* [3]*Laboratoire de Biologie des Tumeurs and* [4]*Département d'Oncology, Médicale Institut Paoli-Calmettes, 232 Boulevard de Ste-Marguerite, 13273 Marseille Cédex 9, France.*

**Summary** The expression of p53 protein was examined in a series of 136 primary breast carcinomas, 106 of which were analysed with a panel of four monoclonal antibodies (MAbs 1801, 240, DO7 and DO1). p53 expression was detected with at least one antibody in 40 tumours (38%), whereas only 15 tumours (14%) were positive with all four antibodies. Some variability in the immunostaining could be observed depending on the antibody used. This was noticeable both for the number of positive cells within a section and for the intensity of staining. We therefore selected a panel of 17 tumour sections (nine were highly positive, three with medium to low staining and five with low to negative staining), which we analysed by polymerase chain reaction–single-strand conformation polymorphism analysis (PCR–SSCP) for the presence of a p53 mutation at the molecular level. Mutations were identified in 15 cases. Therefore the proportion of p53-stained cells does not seem to be an exact representation of the number of cancer cells bearing a mutation within a tumour. A statistically significant correlation was observed between p53 expression, regardless of the number of positive antibodies, and grade III disease ($P<0.0001$), oestrogen ($P<0.0001$) or progesterone receptor negativity ($P = 0.0061$), increased Ki 67 index ($P = 0.0018$), epidermal growth factor receptor (EGFR) positivity ($P = 0.0076$) and aneuploidy ($P = 0.037$). No correlation was observed with tumour size or lymph node involvement. In univariate analysis p53 expression was not correlated with disease-free survival, in contrast to the classical prognostic parameters, which were statistically correlated. In this series p53 expression was not a marker of early recurrence.

The identification of biological and molecular parameters allowing discrimination between subsets of breast cancers is an important challenge for improved management of the disease. Currently, clinical parameters such as TNM staging, metastatic involvement of axillary lymph nodes and histological grading remain the major prognostic factors (Fisher *et al.*, 1975; Contesso *et al.*, 1987). However, biological parameters such as hormone receptor status and DNA ploidy have also proven helpful (Clark *et al.*, 1989; McGuire *et al.*, 1990). More recently, studies on alterations affecting oncogenes and anti-oncogenes have suggested that these may also become interesting prognostic indicators (Slamon *et al.*, 1989; Gullick *et al.*, 1990).

Inactivation or abnormal expression of anti-oncogenes appears to play an important role in the genesis and/or progression of cancer. The wild-type p53 protein possesses growth-suppressing potential and acts as a cell cycle checkpoint, whereas its mutated counterpart either loses its anti-oncogene characteristics or acquires oncogenic potential (Lane & Benchimol, 1990; Kastan *et al.*, 1992). These properties, added to the observation by Nigro *et al.* (1989) that p53 could be mutated in a substantial proportion of colon carcinomas, quickly made this gene the most studied topic in human cancer. p53 turned out to be the most frequently altered gene in human tumours, since all the cancer types analysed to date have displayed p53 mutations, although at varying frequencies (Hollstein *et al.*, 1991). An interesting characteristic of p53 mutations (reviewed in Caron de Fromentel & Soussi, 1992) lies in the increased stability of the mutated protein, whose half-life is increased by a factor of 10–20 as compared with the wild-type protein (Lane & Benchimol, 1990). This leads to the accumulation of mutated p53 protein in the cell nucleus, which makes it detectable by standard immunostaining procedures (Lane & Benchimol, 1990). This property has prompted a large number of studies on p53 protein expression in various tumours, including breast carcinomas (Catoretti *et al.*, 1988; Bartek *et al.*, 1990*a*, *b*).

It was quickly found out that p53 immunoreactivity could bear some prognostic significance. Mutated or overexpressed p53 was correlated with markers of poor prognosis such as high histopathological grading, high levels of Ki 67 or EGF receptor, absence of hormone receptors, as well as with a shortened disease-free or overall survival (Catoretti *et al.*, 1988; Davidoff *et al.*, 1991*a*; Horak *et al.*, 1991; Iwaha *et al.*, 1991; Ostrowski *et al.*, 1991; Barbareschi *et al.*, 1992; Isola *et al.*, 1992; Poller *et al.*, 1992; Thor *et al.*, 1992; Allred *et al.*, 1993; Barnes *et al.*, 1993; Silvestrini *et al.*, 1993). However, variations can be found from one study to another concerning the frequencies of positively stained tumours, which vary from 20 to 55%. Patterns of immunoreactivity also show some variability, since staining could either be purely nuclear or fully cytoplasmic (Horak *et al.*, 1991). It has, furthermore, been noticed that the frequency of p53 mutants detected by immunostaining is often twice as high as by polymerase chain reaction (PCR)-based methods such as single-strand conformation polymorphism (SSCP), denaturation gradient gel electrophoresis (DGGE) or chemical cleavage (Mazars *et al.*, 1992).

These elements led us to evaluate the detection of p53 accumulation in a series of breast tumours for which sections could be made from both frozen and formalin-fixed/paraffin-embedded tissues. In the present work, we analysed p53 immunostaining using four monoclonal antibodies on frozen and/or fixed tissues, and calculated its prognostic significance. In addition, we cross-correlated the presence of a mutation at the molecular level by performing a PCR–SSCP analysis on a selected set of positively stained sections.

### Materials and methods

### *Patients and tumour samples*

A series of 136 patients with breast carcinoma diagnosed between January 1988 and December 1992 was analysed. The

Correspondence: J. Jacquemier, Institut Paoli-Calmettes, Département de Pathologie, 232 Bd Sainte Marguerite, 13273 Marseille cedex 9, France.
Received 6 August 1993; and in revised form 3 December 1993.

median follow-up of these patients was short, 10.7 months (range 6 months to 4 years). Breast cancer recurrences were observed in 12 patients and four patients died from metastasis during the follow-up period. Most of the patients (97) were post-menopausal (older than 50 years old). Tumours varied in size at the time of diagnosis: subclinical tumours, 5%; T1, 15%; T2, 60%; T3, 15%; T4, 5%. Forty-eight per cent (62) of the patients presented axillary lymph node invasion and received adjuvant chemotherapy. Most of the cancers analysed were invasive ductal carcinoma. However, eight were infiltrating lobular and five were of the tubular type.

A portion of each tumour was snap frozen in liquid nitrogen and stored at −70°C for immunohistochemistry and flow cytometry, or fixed in buffered formalin for paraffin embedding. Several biological parameters were analysed by immunohistochemistry simultaneously with p53.

### *p53 antibodies*

Four different commercially available monoclonal anti-p53 antibodies were used. Their characteristics are described in Table I. PAb 1801 was used on 136 tumours, while the three other antibodies were applied to 106 tumours.

### *Immunohistochemistry*

The frozen sections were air dried for 10 min and fixed in acetone at 18°C for 10 min. The paraffin sections were pretreated three times by microwaving at 700 W in citrate buffer pH 6.2 for 15 min. After standard inhibition of endogenous peroxidase activity, staining of p53 protein was performed with the different monoclonal antibodies under the conditions detailed in Table I. Binding of the antibodies was detected by using the ABC immunoperoxidase system (Vectastain, Vector). All series included positive controls. Negative controls included substitution of monoclonal antibody by phosphate-buffered saline (PBS). All controls gave satisfactory results. In addition to p53 antibodies, immunohistochemistry was performed for most of the patients (see Table III) on frozen sections using different antibodies directed against oestrogen and progesterone receptors (Abbott Kit, Rungis, France), EGF receptor (mouse monoclonal antibody EGFR1, Amersham, Les Ullis, France), Ki 67 (mouse monoclonal antibody, Dakopatts, Trappes, France). Results were evaluated by a semiquantitative method (Jacquemier *et al.*, 1986). The p53 protein was revealed by a nuclear staining, and quantitated in terms of percentage of positive cells.

### *Flow cytometry*

Tumour ploidy and S-phase were analysed by FACscan (Beckton Dickinson) on frozen–thawed fragments stained with propidium iodide. The coefficient of variation was below 5% for all statistical analyses.

### *PCR–SSCP analysis*

PCR conditions, pairs of primers used, and SSCP conditions were as previously described (Mazars *et al.*, 1992) except for amplification of sequences in exon 4, which were as follows: A, TGCACCAGCAGCTCCTACAC; B, CATGGAAGCCAGCCCCTCAG. The intensity ratios of mutated *vs* wild-type bands were evaluated by densitometry scanning of the autoradiograms. Autoradiograms were processed on a visage system image analysis workstation (Millipore, Bedford, MA, USA) and quantified.

### *Statistical analysis*

Evaluation of the data was performed using an IBM-compatible statistical analysis package program (Medlog Logisoft). The probability of disease-free survival was calculated for patients using the life table method and generalised salvage (Mantel–Cox) using the BMDP statistical software. Chi-square tests were used to compare levels of p53 protein expression and several other prognostic factors: tumour size, hormone receptor status, ploidy and lymph node status.

### Results

### *Immunohistochemistry assays of p53 protein*

We analysed 136 primary breast carcinomas for p53 staining by immunohistochemistry. For this purpose we used four commercially available antibodies, MAbs 1801, 240, DO7 and DO1 (Table I). In most positive cases, nuclear staining was observed with all four monoclonal antibodies. However, MAb 240 could occasionally give a cytoplasmic staining, which we did not take into account in the absence of concomitant nuclear staining. The intensity of staining varied from one tumour to another but was generally homogeneous within a given sample. No positive staining could be observed in normal mammary tissue. Only the percentage of positive cells was taken into account for statistical analysis, independently of the variation in intensity.

The numbers of positively staining tumours varied depending on the antibody used: MAbs 1801, 240, DO7 and DO1 gave 20%, 24.5%, 20.1% and 29% positive tumours respectively (Table I). Within a section, differences were also seen in the proportions of p53-positive cells. These ranged from 29% with MAb 1801 to 51% with MAb DO1. Overall, 40 tumours (38%) presented positive immunostaining with at least one antibody. Of these, 15 were positive with all four antibodies, nine with a combination of three antibodies, six with two antibodies and ten with only one.

### *Correspondence between p53 antibody staining and PCR–SSCP analysis*

These differences between antibodies in the numbers of positive tumours and in the proportions of stained cells led

**Table I** Characteristics of the four monoclonal antibodies used, percentage of positive cells and positive cases for each antibody

| *Monoclonal antibody* | *Binding site (amino acids)* | *Source* | *Condition of utilisation* | *Number of cases* | *Positive cases* | *Mean percentage of positive cells*[a] |
|---|---|---|---|---|---|---|
| MAb 1801 | 32–79 | Oncogene Science Clinisciences, France | Frozen sections, 5 μg ml$^{-1}$, 1 h, room temperature | 136 | $n$ = 24 (17%) | 29 |
| MAb 240 | 156–355 | Novocastra | Frozen sections, 1:10 dilution, overnight/4°C | 106 | $n$ = 21 (20%) | 37 |
| MAb DO7 | 1–45 | Novocastra TEBU, France | Frozen sections, 1:100 dilution, 1 h, room temperature | 106 | $n$ = 26 (24.5%) | 45 |
| MAb DO1 | 1–45 | Immunotech, Marseille, France | Paraffin sections, 1:300 dilution, 1 h, room temperature after microwave pretreatment | 106 | $n$ = 31 (29%) | 51 |
| All combined | | | | 106 | $n$ = 40 (38%) | |

[a]Not significant with chi-square test.

us to test for the presence of a mutation at the DNA level. This was performed using the PCR-SSCP method in a panel of tumour sections presenting positive immunostaining. Sections to be analysed at the molecular level were selected according to the number of cells stained with the aim of setting up a panel of samples showing strong, medium and low positivity (Table II). Stained tissue sections were scraped, processed for DNA extraction and sequences within exons 4-9 of the p53 gene were amplified by PCR. Radiolabelled PCR fragments were subsequently analysed by SSCP and mutated DNA sequences identified because of their variant electrophoretic patterns. Although the method would not apply for exact quantification, we found that it could be helpful to estimate the ratio of mutated versus wild-type p53 sequence in a given DNA sample. To verify this, we performed a PCR-SSCP analysis on a cloned mutant p53 sequence serially diluted with a cloned wild-type sequence. As shown in Figure 1, the relative intensities of the mutated and wild-type bands were proportional to the input of their templates. We then performed a PCR-SSCP analysis on 17 sections, of which nine were highly positive, three showed medium to low staining and five low to negative staining (Table II). It is noticeable, however, that most sections displayed different intensities of staining depending on the antibody used (Figure 2). Only in a minority of sections did we find similar staining intensities with all four antibodies. Mutations were identified in 15 of 17 cases, and the relative intensities of the shifted SSCP bands were evaluated as described in Materials and methods, and compared with the immunostaining results (Table II). Whereas a number of tumours presented concordant results with immunohistochemistry and SSCP, this was not generally the case. Sections showing low levels of immunoreactivity could present highly prevalent mutated bands on the SSCP gel (sample 5 in Table

**Figure 1** Sensitivity of p53 mutation detection by PCR-SSCP in a dilution test. Wild-type and mutated p53 template DNAs (CMVp53wt plasmid and CMVp53aal56 for the mutant) were mixed with increasing dilution ratios as indicated on the top of the figure and subsequently amplified in a radioactive PCR experiment. Radiolabelled PCR fragment were subjected to SSCP analysis and the mutated and wild-type single-stranded conformers characterised according to their migration patterns. DS indicates the position of the double-stranded molecule.

**Figure 2** Comparison of p53 immunohistochemical staining and the PCR-SSCP patterns. Each panel shows on the left side p53 tumour section staining and on the right the corresponding PCR-SSCP experiment performed on DNA extracted from a serial section. **a**, Sample 6 stained with MAb DO1 showing 40% positive cells; the mutated/wild-type sequences ratio was less then 25%. **b**, Sample 5 stained with MAb DO7 revealing 10% positive cells, whereas the mutated/wild-type ratio was higher than 75%. **c**, Sample 8 stained with MAb DO1 showing 5% positive cells and a ratio of 50-75%. **d**, Sample 2 stained with MAb DO7 showing 80% positive cells, whereas the ratio was 50-75%. Mutated conformers are indicated by arrows; lane T corresponds to tumour DNA and WT to a normal control DNA. Mutated/wild-type band intensity ratios were evaluated as described in Materials and methods.

**Table II** p53 expression in 17 cases of breast cancer detected by immunohistochemistry and comparison with SSCP analysis

| *Sample* | *SSCP analysis* | | *Immunohistochemistry analysis*[a] | | | |
|---|---|---|---|---|---|---|
| *no.* | *Exon* | *Intensity*[b] | *MAb 1801* | *MAb DO1* | *MAb DO7* | *MAb 240* |
| 1 | 4 | ++ | 80 | 40 | 70 | 0 |
| 2 | 4 | +++ | 80 | 50 | 80 | 0 |
| 3 | 4 | ++ | 0 | 0 | 0 | 20 |
| 4 | 5 | ++++ | 80 | 20 | 80 | 20 |
| 5 | 5 | ++++ | 1 | 70 | 10 | 20 |
| 6 | 5 | + | 1 | 40 | 20 | 0 |
| 7 | 5′ | +++ | 30 | 70 | 80 | 40 |
| 8 | 5′ | +++ | 0 | 5 | 2 | 0 |
| 9 | 5′ | ++ | 0 | 40 | 1 | 0 |
| 10 | 5′ | + | 5 | 40 | 1 | 5 |
| 11 | 6 | ++ | 80 | 80 | 80 | 80 |
| 12 | 6 | ++ | 60 | 80 | 80 | 60 |
| 13 | 8 | + | 30 | 0 | 20 | 0 |
| 14 | 8 | ++ | 30 | 80 | 80 | 80 |
| 15 | 8 | ++ | 20 | 80 | 70 | 30 |
| 16 | – | – | 40 | 60 | 60 | 60 |
| 17 | – | – | 5 | 40 | 10 | 30 |

[a]Percentage of cells stained with the different antibodies. [b]Relative intensity of the shifted bands. +, <25%; ++, <50%; +++, <75%; ++++, >75%.

**Table III** Correlation between the different prognostic criteria studied and the expression of p53 protein expression with a minimum of one positive antibody

| *Criterion* | *Number of cases* | *p53 expression Number of positive cases* | *p53 expression Percentage of positive cases* | $\chi^2$ | *P* |
|---|---|---|---|---|---|
| Tumour size | | | | | |
| <20 mm | 40 | 11 | 28 | 0.029 | 0.86 |
| ≥20 mm | 91 | 28 | 72 | | |
| ND | 6 | | | | |
| Histological grade | | | | | |
| I | 18 | 2 | 5 | | |
| II | 74 | 14 | 36 | 21.96 | <0.0001 |
| III | 40 | 23 | 59 | | |
| ND | 5 | | | | |
| Lymphoid stromal reaction | | | | | |
| 0 | 72 | 15 | 38.5 | | |
| + | 42 | 16 | 41 | 7.23 | 0.026 |
| ++ | 16 | 8 | 20.5 | | |
| ND | 7 | | | | |
| Nodal status | | | | | |
| N = 0 | 66 | 15 | 42 | | |
| N<4 | 34 | 12 | 33 | 2.09 | 0.3 |
| N≥4 | 28 | 9 | 25 | | |
| ND | 9 | | | | |
| ER status | | | | | |
| Negative | 41 | 23 | 57.5 | 18.33 | <0.0001 |
| Positive | 95 | 17 | 42.5 | | |
| ND | 1 | | | | |
| PR status | | | | | |
| Negative | 62 | 26 | 65 | 7.5 | 0.0061 |
| Positive | 74 | 14 | 35 | | |
| ND | 1 | | | | |
| Ki 67 | | | | | |
| <15% | 77 | 16 | 42 | 9.78 | 0.0018 |
| ≥15% | 44 | 22 | 58 | | |
| ND | 16 | | | | |
| EGFR | | | | | |
| Negative | 99 | 24 | 69 | 7.11 | 0.0076 |
| Positive | 19 | 11 | 31 | | |
| ND | 19 | | | | |
| Ploidy | | | | | |
| Diploid | 45 | 7 | 18 | 6.5 | 0.037 |
| Aneuploid | 84 | 31 | 82 | | |
| ND | 8 | | | | |

ND, not determined; ER, oestrogen, receptor; PR, progesterone receptor; EGFR, epidermal growth factor receptor.

II) and, conversely, samples with highly positive staining exhibited a mutated/wild-type band ratio of about 50:50 (samples 11 and 12, Table II). We conclude that the proportions of p53-stained cells do not give an exact representation of the number of cancer cells bearing a gene mutation within a tumour.

*Correlation with different biological parameters*

Positive p53 reactivity was tested for correlations with common clinicopathological parameters and with recent prognostic markers such as immunohistochemical measurement of EGFR and Ki 67 levels, as well as ploidy (Table III).

Interestingly, p53 positivity seemed to be dependent on the type of tumour tested, since only infiltrating ductal carcinomas stained positively, whereas tumours of other types scored negative. Statistically significant correlations were found with a number of parameters known to be associated with poor prognosis (Table III). For instance, positive p53 reactivity was strongly correlated with grade III disease ($P = 0.0001$), loss of oestrogen receptor ($P = 0.0001$) or progesterone receptor ($P = 0.0061$) expression, positive Ki 67 staining ($P = 0.0018$) and EGFR expression ($P = 0.0076$). Less significant values were obtained with aneuploidy or lymphoid stromal reaction. These results indicate that positive p53 immunostaining is associated with highly proliferative or aggressive breast carcinoma showing most of the signs of aggressiveness. However, it is interesting to note that no association could be found with axillary lymph node involvement. These results were independent of the antibody used.

*p53 expression and clinical evolution of the disease*

In order to assess the prognostic value of positive p53 immunostaining and compare it with that of usual markers of prognosis, we performed an univariate analysis (Table IV). The results indicate that, in the presently studied set of patients, the classical prognostic parameters were correlated with disease-free survival such as tumour size ($P = 0.043$), nodal status ($P = 0.0030$), oestrogen receptor ($P = 0.0108$) and progesterone receptor ($P = 0.011$).

In contrast, histoprognostic grade and p53 staining were not correlated with disease-free survival for this short period of follow-up. This absence of correlation was observed whatever the antibody used.

### Discussion

Abnormal expression of p53 protein has been widely studied in human breast cancer, and most investigators conclude an association with aggressive forms of the disease (Iwaya *et al.*, 1991; Ostrowski *et al.*, 1991; Isola *et al.*, 1992; Thor *et al.*, 1992). However, important differences can be found from one study to another, the number of positively stained samples ranging from 22% to more than 50%. In the present study, we used a combination of four commercially available monoclonal antibodies to assess the accumulation of p53 protein in a series of 136 primary breast cancers. We observed 38% of the tumours staining positively with at least one antiserum. Numbers were lower when each antibody was considered individually, ranging from 17% to 30%. Our

**Table IV** Univariate analysis of actuarial disease-free survival

| *Criterion* | *Number of cases* | *Disease-free survival (%) Two years* | *Three years* | *P* |
|---|---|---|---|---|
| Tumour size | | | | |
| <20 mm | 40 | – | – | 0.043 |
| ⩾20 mm | 91 | 87 | 79 | |
| ND | 6 | | | |
| Histological grade | | | | |
| I | 18 | – | – | |
| II | 74 | 91 | 81 | 0.45 |
| III | 40 | 85 | 85 | |
| ND | 5 | | | |
| Nodal status | | | | |
| Negative | 66 | – | – | 0.003 |
| Positive | 62 | 84 | 73 | |
| ND | 9 | | | |
| ER status | | | | |
| Negative | 41 | 78 | 62 | 0.0108 |
| Positive | 95 | 94 | 91 | |
| ND | 1 | | | |
| PR status | | | | |
| Negative | 62 | 80 | 69 | 0.0112 |
| Positive | 74 | 96 | 92 | |
| ND | 1 | | | |
| p53 status[a] | | | | |
| Negative | 66 | 90 | 87 | 0.17 |
| Positive | 40 | 85 | 64 | |
| ND | 31 | | | |
| PAb 1801[b] | | | | |
| Positive | 24 | 90 | 60 | 0.35 |
| PAb 240[b] | | | | |
| Positive | 21 | 87 | 87 | 0.75 |
| DO1[b] | | | | |
| Positive | 31 | 86 | 65 | 0.19 |
| DO7[b] | | | | |
| Positive | 26 | 92 | 69 | 0.54 |
| ND | | | | |

[a]Global status with four antibodies. [b]By comparison with the 66 cases negative with four antibodies.

numbers are in agreement with those reported by Thor *et al.* (1992), but remain low compared with those reported by a number of other investigators (Cattoretti *et al.*, 1988; Bartek *et al.*, 1991; Horak *et al.*, 1991). Variations might reflect heterogeneity between the tested cohorts of tumours, as testified by the larger numbers of p53-positive cases observed in Li–Fraumeni patients compared with a population of sporadic breast cancers (Thor *et al.*, 1992), but technical conditions could also account for these disparities. In fact, the conditions of fixation (type of fixative and duration of the process) and antibody used have been shown to influence greatly the numbers of positive cases and the patterns of staining, such as the numbers of stained cells and cytoplasmic positivity (Bartek *et al.*, 1990*a*, *b*). Some of our own observations (data not shown), as well as variations in the proportions of tumour cells showing immunoreactivity when different antibodies were compared – some tumours could show 70 or 80% positive cells with a given MAb and be negative or weakly positive with another antiserum – confirm that p53 immunostaining depends greatly on the conditions and reagents used in the test. Our data indicate that using several antibodies in combination may reduce the extent of the variations. Results by Varley *et al.* (1991) and Allred *et al.* (1993) further support this observation.

These variations in staining patterns as well as reports on possible discrepancies between immunohistochemistry and analyses for mutations at the DNA level (Borresen *et al.*, 1991; Thompson *et al.*, 1992) led us to perform a comparative search for mutations in the p53 gene using PCR–SSCP on a selected set of p53-positive breast tumours. Our data suggest that there is a good overlap between the presence of a mutation at the DNA level and positive immunostaining in breast cancer since only 2/17 samples scored negative upon PCR–SSCP analysis. The correspondence might even be better given the fact that mutations may be missed by PCR–SSCP, as shown by some of our comparative tests with DGGE (C. Moyret, C. Theillet, L. Puig, J.P. Molès, G. Thomas & R. Hamelin, submitted). Therefore, the two tumours in which no mutation was detected despite the presence of positive staining may still bear a mutated p53 allele. If, according to the present data, positive immunostaining often corresponds to the presence of a mutation at the genetic level, the reverse is not always true. We used an inverse approach by testing by immunohistochemistry tumours which we had previously analysed for PCR–SSCP and found that most mutants presented p53 staining; only nonsense mutants were negative (J.P. Molès & C. Theillet, unpublished results). The next question we asked was if the number of cells stained with an anti-p53 antibody within a tumour section reflected the number of cells actually bearing a mutated p53 gene. As a matter of fact, a number of studies have shown that some breast tumours could present focal p53 staining corresponding to islets of p53-positive cells (Bartek *et al.*, 1991; Davidoff *et al.*, 1991*b*; Horak *et al.*, 1991). These results, added to some of our SSCP observations showing that the wild-type allele was frequently retained in tumours presenting mutated p53 sequences (Mazars *et al.*, 1992), suggested to us that the mutation might be present only in a fraction of the cancer cells forming these samples. Therefore, we wanted to verify if the proportions of cancer cells stained by immunohistochemistry were concordant with the signal ratio of mutated to wild-type sequences in the SSCP analyses, thus implying that p53 staining was indicative of the actual number of mutated cells. The results presented here indicate that while some concordance could be found some disparities also existed. Hence, we conclude that p53 staining is a somewhat imperfect reflection of the prevalence of p53 mutation and this is, in our mind, further supported by differences in staining when different antibodies are compared. Furthermore, heterogeneity in staining might be increased by physiological responses such as stabilisation of mild p53 in response to a genotoxic stress.

Positive p53 immunostaining has, in a number of reports, been correlated with factors associated with poor prognosis, early relapse or shortened disease-free survival (Thor *et al.*, 1992; Allred *et al.*, 1993). Only two studies did not show similar correlations (Varley *et al.*, 1991; Poller *et al.*, 1992). Most studies show a correlation in either monoparametric (Iwaya *et al.*, 1991; Ostrowski *et al.*, 1991; Isola *et al.*, 1992; Thor *et al.*, 1992) or multiparametric analysis. In our series such a correlation could not be observed. This could be attributed to a short follow-up. In contrast, most classical parameters, such as nodal status or oestrogen and progesterone receptors, were significantly correlated. This suggests that, in this series, p53 detected by immunohistochemistry is not indicative of early recurrence.

Despite the fact that immunostaining does not permit the detection of all mutations affecting p53 (nonsense mutations do not lead to p53 accumulation), recent reports showing the possibility of p53 staining in the absence of detectable mutations are an additional argument in favour of this method in the assessment of high-risk breast cancer patients. However, the variations in staining patterns and numbers of positive tumours one observed depending on the antibody and/or technical conditions used necessitates standardisation of the methods and reagents used in such studies.

The authors thank Mrs M. Clavelly and C. Giorgetti for manuscript preparation and Dr J. Gouvernet for statistical analysis.

This work was supported by the Paoli-Calmettes Cancer Institute and by les Comités de Bouches des bouches du Rhône et du Var de 'La Ligue Departementale Contre le Cancer'.

### References

ALLRED, D.C., CLARK, G.M., ELLEDGE, R., FUQUA, S.A.W., BROWN, R.W., CHAMNESS, G.C., OSBORNE, C.K. & MCGUIRE, W.L. (1993). Association of p53 protein expression with tumour cell proliferation rate and clinical outcome in node-negative breast cancer. *J. Natl Cancer Inst.*, **85**, 200–206.

BARBARESCHI, M., LEONARDI, E., MAURI, F.A., SERIO, G. & DALLA PALMA, P. (1992). p53 and *erb*B-2 protein expression in breast carcinomas; an immunohistochemical study including correlations with receptor status, proliferation markers, and clinical stage in human breast cancer. *Am. J. Clin. Pathol.*, **98**, 408–418.

BARNES, D.M., DUBLIN, E.A., FISHER, C.J., LEVISON, D.A., MILLIS, R.R. (1993). Immunohistochemical detection of p53 protein in mammary carcinoma. An important new independent indicator of prognosis? *Hum. Pathol.*, **24**, 469–476.

BARTEK, J., IGGO, R., GANNON, J. & LANDE, D.P. (1990*a*). Genetic and immunohistochemical analysis of mutant p53 in human breast cancer cell lines. *Oncogene*, **5**, 893–899.

BARTEK, J., BARTKOVA, J., VOJTESEK, B., STASKOVA, Z., REJTHAR, A., KOVARIK, J. & LANE, D. (1990*b*). Patterns of expression of the p53 tumour suppressor in human breast tissues and tumours *in situ* and *in vitro*. *Int. J. Cancer*, **46**, 839–844.

BARTEK, J., BARTKOVA, J., VOJTESEK, B., STASKOVA, Z., LUKAS, J., REJTHAR, A., KOVARIK, J., MIDGLEY, C.A., GANNON, J.V. & LANE, D.P. (1991). Aberrant expression of the p53 oncoprotein is a common feature of a wide spectrum of human malignancies. *Oncogene*, **6**, 1699–1703.

BORRESEN, A.L., HOVIG, E., SMITH-SORENSEN, B., MALKIN, D., LYSTAD, S., ANDERSEN, T.I., NESLAND, J.M., ISSELBACHER, K.J. & FRIEND, S.H. (1991). Constant denaturant gel electrophoresis as a rapid screening technique for p53 mutations. *Proc. Natl Acad. Sci. USA*, **88**, 8405–8409.

CARON DE FROMENTEL, C. & SOUSSI, T. (1992). TP53 tumor suppressor gene: a model for investigating human mutagenesis. *Genes. Chrom. Cancer*, **4**, 1–15.

CATTORETTI, G., RILKE, F., ANDREOLA, S., D'AMATO, L. & DELIA, D. (1988). P53 expression in breast cancer. *Int. J. Cancer*, **41**, 178–183.

CLARK, G.M., DRESSLER, L.G., OWENS, M.A., POUNDS, G., OLDAKER, T. & MCGUIRE, W.L. (1989). Prediction of relapse or survival in patients with node-negative breast cancer by DNA flow cytometry. *N. Engl. J. Med.*, **320**, 627–633.

CONTESSO, G., MOURIESSE, H., FRIEDMAN, S., GENIN, J., SARRAZIN, D. & ROUESSE, J. (1987). The importance of histologic grade in long-term prognosis of breast cancer: a study of 1,010 patients, uniformly treated at the institut Gustave-Roussy. *J. Clin. Oncol.*, **5**, 1378–1386.

DAVIDOFF, A., KERNS, B.J., PENCE, J., MARKS, J. & IGLEHART, D. (1991*a*). p53 alterations in all stages of breast cancer. *J. Surg. Oncol.*, **48**, 260–267.

DAVIDOFF, A.M., HUMPHREY, P.A., IGLEHART, J.D. & MARKS, J.R. (1991*b*). Genetic basis for p53 overexpression in human breast cancer. *Proc. Natl Acad. Sci. USA*, **88**, 5006–5010.

FISHER, E.R., GREGORIO, R.M. & FISHER, B. (1975). The pathology of invasive breast cancer: a syllabus derived from findings of National Surgical Adjuvant Breast Project (protocol no. 4). *Cancer*, **36**, 1–85.

GULLICK, W.J. (1990). The role of the epidermal growth factor receptor and the c-*erb*B-2 protein in breast cancer. *Int. J. Cancer*, **5** (Suppl.), 55–61.

HOLLSTEIN, M., SIDRANSKY, D., VOGELSTEIN, B. & HARRIS, C.C. (1991). p53 mutations in human cancers. *Science*, **253**, 49–53.

HORAK, E., SMITH, K., BROMLEY, L., LEJEUNE, S., GREENALL, M., LANE, D. & HARRIS, A. (1991). Mutant p53, EGF receptor and c-*erb*B-2 expression in human breast cancer. *Oncogene*, **6**, 2277–2284.

ISOLA, J., VISAKORPI, T., HOLLI, K. & KALLIONIEMI, O.P. (1992). Association of overexpression of tumor suppressor protein p53 with rapid cell proliferation and poor prognosis in node-negative breast cancer patients. *J. Natl Cancer Inst.*, **84**, 1109–1114.

IWAYA, K., TSUDA, H., HIRAIDE, H., TAMAKI, K., TAMAKUMA, S., FUKOTOMI, T., MUKAI, K. & HIROHASHI, S. (1991). Nuclear p53 immunoreaction associated with poor prognosis of breast cancer. *J. Cancer Res.*, **82**, 835–840.

JACQUEMIER, J., CHARPIN, C. & MARTIN, P.M. (1986). Etude immunohistochimique par anticorps monoclonal (H222 SPγ) des récepteurs oestrogéniques: corrélations avec l'analyse biochimique par radioligand pour 115 carcinomes mammaires. *Bull. Cancer*, **73**, 107–119.

KASTAN, M.B., ZHAN, Q., EL-DEIRY, W.S., CARRIER, F., JACKS, T., WALSH, W.V., PLUNKETT, B.S., VOGELSTEIN, B. & FORNACE, Jr, A.J. (1992). A mammalian cell cycle checkpoint pathway utilizing p53 and *GADD45* is defective in ataxia-telangiectasia. *Cell*, **71**, 587–597.

LANE, D. & BENCHIMOL, S. (1990). p53: oncogene or anti-oncogene. *Genes Dev.*, **4**, 1–8.

MCGUIRE, W.L., TANDON, A.K., ALLRED, D.C. & others (1990). How to use prognostic factors in axillary node-negative breast cancer patients. *J. Natl Cancer Inst.*, **82**, 1006–1015.

MAZARS, R., SPINARDI, L., BENCHEIKH, M., SIMONY-LAFONTAINE, J., JEANTEUR, P. & THEILLET, C. (1992). *p53* mutations occur in aggressive breast cancer. *Cancer Res.*, **52**, 3918–3923.

NIGRO, J.M., BAKER, S.J., PREISINGER, A.C., JESSUP, J.M., HOSTETTER, R., CLEARY, K., BIGNER, S.H., DAVIDSON, N., BAYLIN, S., DEVILEE, P., GLOVER, T., COLLINS, F.S., WESTON, A., MODALI, R., HARRIS, C.C. & VOGELSTEIN, B. (1989). Mutations in the p53 gene occur in diverse human tumour types. *Nature*, **342**, 705–708.

OSTROWSKI, J.L., SAWAN, A., HENRY, L., WRIGHT, C., HENRY, J.A., HENNESSY, C., LENNARD, T.J., ANGUS, B. & HORNE, C.H. (1991). p53 expression in human breast cancer related to survival and prognostic factor: an immunohistochemical study. *J. Pathol.*, **164**, 75–81.

POLLER, D.N., HUTCHINGS, C.E., GALEA, M., BELL, J.A., NICHOLSON, R.A., ELSTON, C.W., BLAMEY, R.W. & ELLIS, I.O. (1992). p53 protein expression in human breast carcinoma: relationship to expression of epidermal growth factor receptor, c-*erb*B-2 protein overexpression, and oestrogen receptor. *Br. J. Cancer*, **66**, 583–588.

SILVESTRINI, R., BENINI, E., DAIDONE, M.G., VENERONI, S., BORACCHI, P., CAPPELLETTI, V., DI FRONZO, G. & VERONESI, U. (1993). p53 as an independent prognostic marker in lymph node-negative breast cancer patients. *J. Natl Cancer Inst.*, **85**, 965–970.

SLAMON, D.J., CLARK, G.M., WONG, S.G., LEVIN, W.J., ULLRICH, A. & MCGUIRE, W.L. (1987). Human breast cancer: correlation of relapse and survival with amplification of the HER-2/*neu* oncogene. *Science*, **235**, 177–182.

THOMPSON, A.M., ANDERSON, T.J., CONDIE, A., PROSSER, J., CHETTY, U., CARTER, D.C., EVANS, H.J. & STEEL, C.M. (1992). p53 allele losses, mutations and expression in breast cancer and their relationship to clinico-pathological parameters. *Int. J. Cancer*, **50**, 528–532.

THOR, A.D., MOORE II, D.H., EDGERTON, S.M., KAWASAKI, E.S., REIHSAUS, E., LYNCH, H.T., MARCUS, J.N., SCHWARTZ, L., CHEN, L.C., MAYALL, B.H. & SMITH, H.S. (1992). Accumulation of p53 tumor suppressor gene protein: an independent marker of prognosis in breast cancers. *J. Natl Cancer. Inst.*, **84**, 845–855.

VARLEY, J.M., BRAMMAR, W.J., LANE, D.P., SWALLOW, J.E., DOLAN, C. & WALKER, R.A. (1991). Loss of chromosome 17pl3 sequences and mutation of p53 in human breast carcinomas. *Oncogene*, **6**, 413–421.